# Diversity in *lac* Operon Regulation among Diverse Escherichia coli Isolates Depends on the Broader Genetic Background but Is Not Explained by Genetic Relatedness

**DOI:** 10.1128/mBio.02232-19

**Published:** 2019-11-12

**Authors:** Kelly N. Phillips, Scott Widmann, Huei-Yi Lai, Jennifer Nguyen, J. Christian J. Ray, Gábor Balázsi, Tim F. Cooper

**Affiliations:** a Department of Biology and Biochemistry, University of Houston, Houston, Texas, USA; b School of Natural and Computational Science, Massey University, Auckland, New Zealand; c Center for Computational Biology and Department of Molecular Biosciences, University of Kansas, Lawrence, Kansas, USA; d Laufer Center for Physical and Quantitative Biology and Department of Biomedical Engineering, Stony Brook University, Stony Brook, New York, USA; University of Pittsburgh; Georgia Institute of Technology School of Biological Sciences

**Keywords:** *lac* operon regulation

## Abstract

Transcription of bacterial genes is controlled by the coordinated action of *cis-* and *trans*-acting regulators. The activity and mode of action of these regulators can reflect different requirements for gene products in different environments. A well-studied example is the regulatory function that integrates the environmental availability of glucose and lactose to control the Escherichia coli *lac* operon. Most studies of *lac* operon regulation have focused on a few closely related strains. To determine the range of natural variation in *lac* regulatory function, we introduced a reporter construct into 23 diverse E. coli strains and measured expression with combinations of inducer concentrations. We found a wide range of regulatory functions. Several functions were similar to the one observed in a reference lab strain, whereas others depended weakly on the presence of cAMP. Some characteristics of the regulatory function were explained by the genetic relatedness of strains, indicating that differences varied on relatively short time scales. The regulatory characteristics explained by genetic relatedness were among those that best predicted the initial growth of strains following transition to a lactose environment, suggesting a role for selection. Finally, we transferred the *lac* operon, with the *lacI* regulatory gene, from five natural isolate strains into a reference lab strain. The regulatory function of these hybrid strains revealed the effect of local and global regulatory elements in controlling expression. Together, this work demonstrates that regulatory functions can be varied within a species and that there is variation within a species to best match a function to particular environments.

## Abstract (precis)

The *lac* operon of Escherichia coli is a classic model for studying gene regulation. This study has uncovered features such as the environmental input logic controlling gene expression, as well as gene expression bistability and hysteresis. Most *lac* operon studies have focused on a few lab strains, and it is not known how generally those findings apply to the diversity of E. coli strains. We examined the environmental dependence of *lac* gene regulation in 20 natural isolates of E. coli and found a wide range of regulatory responses. By transferring *lac* genes from natural isolate strains into a common reference strain, we found that regulation depends on both the *lac* genes themselves and on the broader genetic background, indicating potential for still-greater regulatory diversity following horizontal gene transfer. Our results reveal that there is substantial natural variation in the regulation of the *lac* operon and indicate that this variation can be ecologically meaningful.

## INTRODUCTION

Gene regulatory networks allow bacteria to respond to changes in their environment by activating or repressing target genes (1). In this way, cells can exhibit phenotypes that balance the demands of expressing necessary genes while minimizing the diverse costs associated with the expression of genes that are not necessary (2–8). Regulatory networks must respond to a diverse array of signals, for example, integrating information regarding the availability of multiple resources that the organism uses with different preference (9). For a particular group of coregulated genes, the integration of these signals defines its regulatory input function. Knowledge of this function aids in the prediction of gene responses, understanding of the mechanistic basis of regulation, and understanding of the potential for regulation to evolve, and it is likely to be helpful in the pursuit of engineering of specific responses in artificial circuits (10). Despite the importance of regulatory input functions, the understanding of their variation within a species is limited. This variation is important, as it reflects the potential for evolutionary changes in regulatory function and might reveal differences in selection pressures affecting different subpopulations.

A good model system with which to study a regulatory input function is the *lac* operon (*lacZYA*) of Escherichia coli (11–15). This operon has been a focus of efforts to examine the effect on gene expression and regulation of transcription factor stochasticity (16), DNA topology (15), transcriptional fidelity (17), and hysteresis (18). It has also been examined to understand the costs of protein expression (3, 6, 19) and the importance of coordinated gene expression (20) and is established as a target of selection during growth in defined environments (3, 21, 22). The wealth of information gained from empirical study of *lac* operon regulation has made it a focus of attempts to understand and model gene regulation, including attempts to learn how to manipulate the system to change regulatory outputs (14, 20, 23–25).

The *lac* operon encodes three gene products. LacY is a permease that imports lactose into the cell where it is cleaved by LacZ, a β-galactosidase, into glucose and galactose. LacA is a transacetylase that is thought to facilitate the export of toxic sugars that cannot be metabolized by the cell. These genes are beneficial to express in environments where lactose is the best available carbon source, being required for its import and initial catabolism, but their expression is also associated with a significant cost (3, 6, 21). The *lac* operon is directly regulated by two environmental signals, positively by lactose and negatively by glucose, that modulate the activity of transcription factors that bind to *cis*-regulatory DNA regions. The LacI repressor, a *trans*-regulator, binds at three operator binding sites, the *cis*-regulators, in the vicinity of the *lac* promoter and can interact to cause DNA looping, which promotes repressor binding and increases repression (26, 27). In the presence of allolactose (a derivative of lactose) or artificial inducers (e.g., isopropyl β-d-thiogalactoside [IPTG]), LacI is released from DNA, allowing transcription to occur (28). The cAMP-cAMP receptor protein (cAMP-CRP) global regulator complex, another *trans*-acting factor, binds upstream of the *lac* operon promoter to its *cis*-regulatory region and enhances transcription by promoting the recruitment of RNA polymerase to the *lac* promoter (29). The production of cAMP is decreased in the presence of glucose, thereby decreasing the availability of the cAMP-CRP complex.

The regulatory control of many genes can be described as logic functions. These functions integrate complex mechanistic details of regulatory control to describe how regulator activities combine at a *cis*-regulatory region to determine the expression of target genes (13, 14, 30). A simple expectation is that *lac* genes will be controlled by AND-type logic, whereby expression requires the presence of lactose and absence of glucose. In fact, experiments using the artificial IPTG inducer and exogenous cAMP to independently control LacI and CRP activity found that the underlying function is more complex, being intermediate between AND and OR functions (13, 14). That work, however, focused on the gene input function of a single K-12 E. coli strain, MG1655, and close derivatives, which may not be representative of other strains. Though often considered a wild-type strain, MG1655 was isolated in 1922 and during subsequent propagation and storage may have been subject to inadvertent selection that affected the *lac* gene input function (31, 32). Even if the *lac* regulatory function has not changed, it remains unknown if different natural isolate strains demonstrate different functions.

Two factors suggest the potential for variation in a given regulatory function within a species. In the case of the *lac* operon, models and experiments have revealed that many different regulatory functions can evolve through single mutations (14, 21, 33). Second, *lac* regulation can be affected by changes occurring outside its immediate regulatory network. Indeed, in a previous study of E. coli populations evolved in environments containing lactose or combinations of lactose and glucose, changes in *lac* expression evolved that were common and due at least in part to mutations occurring outside the canonical regulatory network (21). Moreover, that work found that the nature of *lac* regulatory changes reflected the selection environment. For example, most populations evolving in an environment that fluctuated daily between glucose and lactose evolved to constitutively express the *lac* genes, whereas populations evolved in the simultaneous presence of glucose and lactose evolved a graded response function, allowing a continuous expression response. Similar findings of selection-dependent changes in gene regulation have been found in populations adapted to chemostat environments (22, 34), during the evolution of a stress response network (35), and inferred from selective benefits of naturally occurring variants controlling the biosynthesis of arginine (36). Although studies have not compared detailed *lac* logic functions of different E. coli strains, *lac* structural gene enzyme activity and fitness effect can vary between isolated *lac* operons (4, 34).

To the extent that there is variation in gene regulatory functions, a key question is the relative contribution of *cis*-regulatory changes that affect the expression of a specific transcriptional unit (i.e., an operon) and *trans*-regulatory changes that have the potential to affect the expression of a regulon potentially containing hundreds of genes (37, 38). This distinction is important because a few *trans*-regulatory changes may allow a large number of key expression changes to evolve relatively quickly, whereas the same expression change occurring through *cis*-regulatory change would take much longer, though perhaps with fewer pleiotropic side effects. The distinction between *cis* and *trans* control of gene regulation is also relevant to the consequences of horizontal gene transfer. If adaptive changes in gene regulation are *cis*-regulatory, they are likely to have fewer antagonistic pleiotropic consequences following transfer to alternative genetic backgrounds, allowing transfer to more genetically divergent recipients.

To examine natural variation in the *lac* regulatory input function, we introduced a green fluorescent protein (GFP) reporter driven by the *lac* promoter and containing the primary (O1) and upstream (O3) LacI repressor and CRP binding sites into 21 divergent natural isolate strains and into two reference lab strains, MG1655 and REL606. We found substantial variation in regulatory functions, which we quantified by fitting a simple regulatory model to the observed expression data. Some aspects of this variation were explained by the genetic relatedness of strains, assessed using phylogenies constructed from core and accessory genes, and from only the *lac* genes. Other parameters varied but without any phylogenetic signal, consistent with them changing on a relatively short time scale. Transfer of a subset of *lac* operons into a common reference strain indicated that at least some of the variation is determined by *trans-*regulators encoded by the recipient strain, not the *cis*-regulatory sequences local to the *lac* genes. To the extent that regulatory functions are influenced by *trans-*regulators that have pleiotropic activity that varies between strains, adaptive changes in gene regulatory functions may be less likely to remain beneficial following horizontal transfer to new strains.

## RESULTS

### *lac* gene input function of natural isolate E. coli strains.

**FIG 1:**
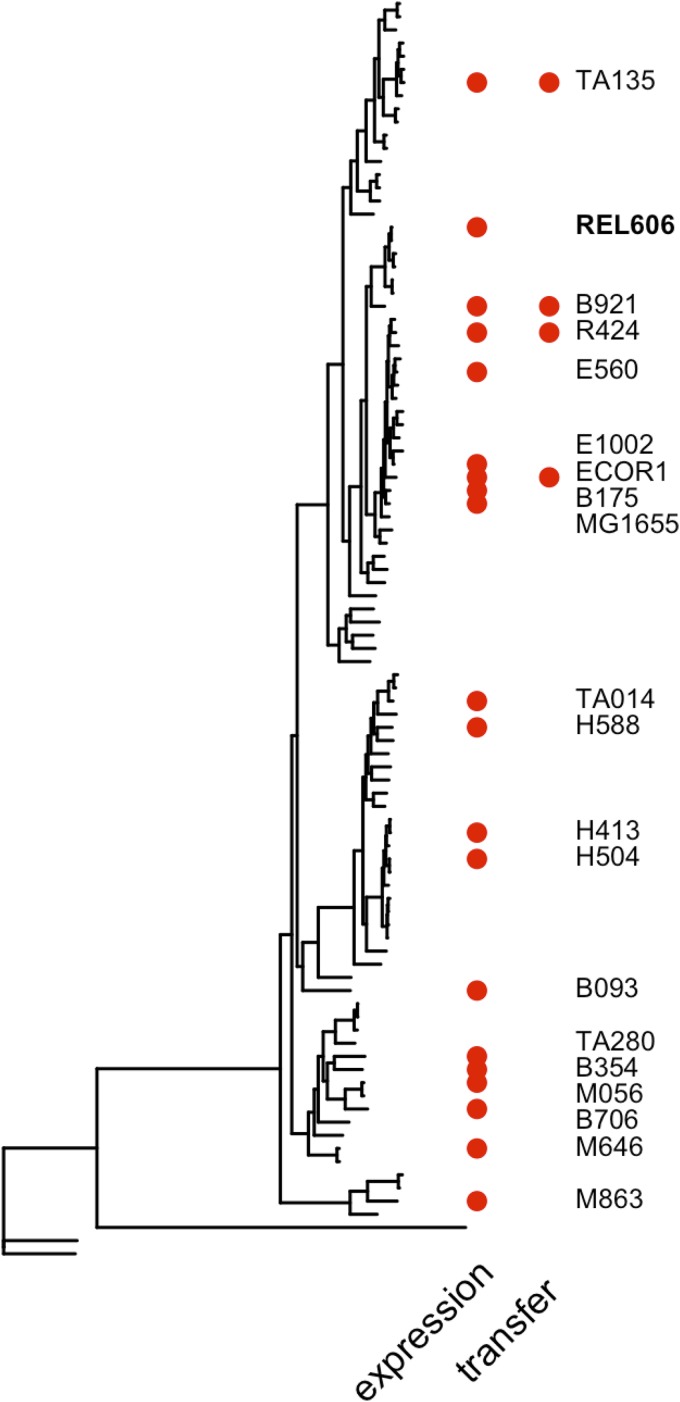
Phylogeny based on the core genome shared between 96 diverse natural isolates of E. coli. Strains whose *lac* regulatory function was determined and whose *lacI-ZYA region* was transferred to the reference strain, REL606, are indicated by the red symbols in columns labeled “expression” and “transfer,” respectively. The former group of strains represents a random sample of the complete phylogeny (Fig. S1). The *lac* regulatory function was also measured for three strains for which we do not have genome sequence and, therefore, are not included here, B156, B1167, and TA263 (Table S1). The *lac* operon of TA263 was also transferred to REL606. Phylogeny construction is described in Materials and Methods.

**FIG 2:**
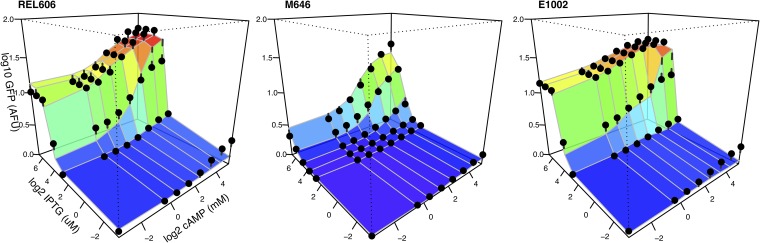
Empirical and modeled gene regulatory profiles. Expression of a *lac* reporter was determined during growth in glucose supplemented with combinations of the inducers cAMP (millimolar) and IPTG (micromolar). Expression was measured from a chromosomally integrated reporter at mid-log phase and is reported in arbitrary fluorescence units (AFU). Solid symbols indicate expression predicted at each measured inducer combination using a simple regulatory model fitted to the observed data (see the supplemental material) (14). Dashed lines connect model estimates and empirically determined expression values. The three profiles shown here are for a lab strain (REL606) and two natural isolate strains (M646 and E1002) and have profiles that differ in the sufficiency of IPTG to induce *lac* expression to a high level. Additional profiles are shown in Fig. S2.

**FIG 3:**
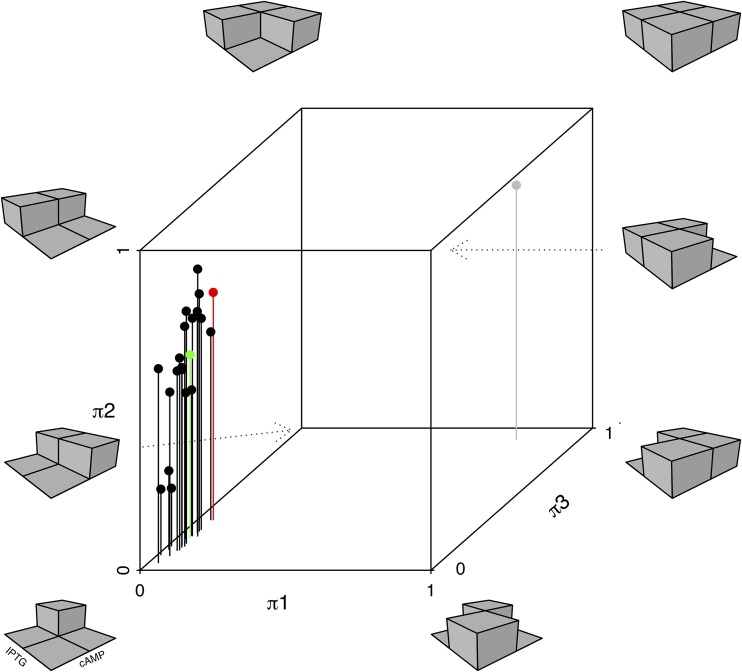
*lac* regulatory logic of E. coli strains. Models describing the *lac* expression phenotype were fitted for each of the tested E. coli strains (Fig. S1). Model parameters were used to determine the ratio of log expression at low IPTG-low cAMP, high IPTG-low cAMP, and low IPTG-high cAMP combinations to the high IPTG-high cAMP combination, giving parameters, π1, π2, and π3, and respectively (14). Combinations of these parameters describe a particular regulatory logic input function. For example, low values of π1, π2, and π3 indicate high *lac* expression only when both IPTG and cAMP levels are present, reflecting an AND-type logic function. Black symbols indicate parameter estimates of natural isolate strains. Green and red points indicate estimates of the lab strains REL606 and MG1655, respectively. The gray point indicates an *E. albertii* strain, B156, that does not encode several components of the canonical lactose utilization system, including a LacI repressor, and therefore expresses the reporter at high levels regardless of IPTG (see also Fig. S2).

We introduced a P*lac*-GFP reporter into 21 natural isolates and two lab strains of E. coli (Fig. 1; see also Table S1 in the supplemental material). Fluorescence from this reporter was measured in combinations of IPTG and cAMP to determine the *lac* operon expression profile of each strain. These profiles exhibit substantial variation across strains (Fig. 2 and S2). We follow two approaches to quantify this variation. First, we fit a simple model to estimate regulatory parameters that explain each strain’s expression profile. This model includes terms corresponding to the interaction of regulatory molecules (IPTG and cAMP) and the transcription factors to which they bind (LacI and CRP, respectively), the activity of those transcription factors, and their interaction with RNA polymerase binding (see Materials and Methods and the Supplemental Text S1 for details) (14). Second, we use the fitted model to infer the regulatory logic function of each response, a measure of the individual and combined effect of cAMP and IPTG inducers on expression (Fig. 3). For example, a requirement of both cAMP and IPTG for *lac* expression represents an AND function, whereas either individual inducer being sufficient for high expression represents an OR function.

10.1128/mBio.02232-19.2 FIG S2 Observed and modeled regulatory functions of 23 strains. Colored landscapes indicate experimentally determined *lac* expression profiles using at least 48 different cAMP-IPTG concentration combinations. Solid symbols and drop lines indicate the predicted expression at each measured point derived from a regulatory model fitted to the observed expression data (Materials and Methods for details). Note that B156 is an *E. albertii* strain that does not encode several components of the canonical lactose utilization system, including the LacI repressor, and therefore expresses the *lac* reporter at high levels regardless of the presence of IPTG. Download FIG S2, PDF file, 0.1 MB. Copyright © 2019 Phillips et al. 2019 Phillips et al. This content is distributed under the terms of the Creative Commons Attribution 4.0 International license.

10.1128/mBio.02232-19.7 TABLE S1 Strains used in this study. Download Table S1, PDF file, 0.1 MB. Copyright © 2019 Phillips et al. 2019 Phillips et al. This content is distributed under the terms of the Creative Commons Attribution 4.0 International license.

10.1128/mBio.02232-19.1 FIG S1 Sampled strains are generally representative of the broader strain collection. The solid black line indicates the distribution of shared branch lengths following 1,000 draws from the broader strain collection (Fig. 1) of the same number of strains used in expression and transfer experiments. The red line indicates the shared branch length of the actual recipient strains. *P* values are calculated as the fraction of bootstrapped samples with a lower branch length than the actual sample. Phylogeny (core or accessory)-mutation combinations are indicated in the title of each panel. Download FIG S1, PDF file, 0.01 MB. Copyright © 2019 Phillips et al. 2019 Phillips et al. This content is distributed under the terms of the Creative Commons Attribution 4.0 International license.

10.1128/mBio.02232-19.1 TEXT S1 Model. Download Text S1, DOCX file, 0.1 MB. Copyright © 2019 Phillips et al. 2019 Phillips et al. This is an open-access article distributed under the terms of the Creative Commons Attribution 4.0 International license.

We find considerable variation in both the fitted model parameters and in the logic function characterizing *lac* operon regulation in the different strains. Considering first the regulatory logic phenotype, we find that, by itself, IPTG causes between 18% and 83% (mean ± SD, 57% ± 17%) of maximum *lac* expression. By comparison, by itself, cAMP causes between 5% and 36% (mean ± SD, 21% ± 8%) of maximum *lac* expression. Synergy between cAMP and IPTG was estimated as the difference in maximum expression observed when both are present to the expression expected based on the product of their individual effects. By this measure, strains depended on the combination of inducers for between −1 and 70% of maximum expression (mean ± SD, 28% ± 21%). Together, these results indicate a range of regulatory logic phenotypes, where some strains depend strongly on both inducers (AND-type logic) and others depend largely on the activity of the LacI repressor. We note that while logic phenotypes omit potentially important aspects of the overall expression phenotype, they nevertheless capture similar relationships among strains, as do the overall expression profiles that are described below (one-tailed Mantel test, *r* = 0.41, *P* = 0.005). Comparing logic functions and the parameterization of the regulatory model fitted to the underlying expression profiles, we find differences in how these descriptions of expression cluster strains (Table S2). Logic and model characterizations were only moderately well correlated, consistent with a mapping whereby the same logic function can be realized by different underlying regulatory parameters (one-tailed Mantel test, *r* = 0.22, *P* = 0.11).

10.1128/mBio.02232-19.8 TABLE S2 Model parameterization for natural isolate and lab strains used in this study. Download Table S2, PDF file, 0.10 MB. Copyright © 2019 Phillips et al. 2019 Phillips et al. This content is distributed under the terms of the Creative Commons Attribution 4.0 International license.

### Comparison of gene input functions to evolutionary distance.

It is of interest to examine whether differences in *lac* regulation have been selected for or whether they represent effectively neutral variation. The ideal test would be to examine differences in the fitness consequences of different *lac* regulation phenotypes in ecologically relevant environments. In practice, however, what constitutes such an environment is not known. Moreover, the effect of *lac* regulation on fitness will be confounded by comparisons across different genetic backgrounds. We therefore follow two complementary approaches to assess the potential for regulatory parameters to have been selected. First, we test the expectation that, if regulatory variation is neutral, differences in estimated parameters will correspond to the underlying strain phylogeny (39). Selected differences may correspond to the phylogeny but are more likely to be driven by different ecological pressures relevant to each strain (40, 41). We have previously found that ecological performance of a subset of strains considered here was not correlated with their phylogenetic relationships, indicating that underlying ecological selection is likely to vary independent of phylogenetic relationships (42). Second, we examine the effect of *lac* operon regulatory parameters on growth following their transfer to an environment where *lac* expression is likely to be influential in determining dynamics.

To test for phylogenetic signal present in regulatory logic and model parameters, we assessed variation in those parameters in the context of phylogenies generated based on the core genome common to all strains, the accessory genome comprising genes present in some but not all strains, and a phylogeny based on the *lacI-ZYA* genes. Phylogenetic signal was assessed using Pagel’s λ, which tests for signal against the null hypothesis of a trait evolving independently of an underlying phylogeny, as would be the case if it varied either neutrally or due to selection pressures that were not correlated to genetic relatedness (43). In most cases, the pattern of regulatory parameter variation was not consistent with any of the tested phylogenies. There were two exceptions to this trend, as follows: the *η* parameter, corresponding to the effect of cAMP-CRP on the binding of RNA polymerase to the *lac* promoter, which exhibited phylogenetic signal over all phylogenies considered; and the *m* parameter, corresponding to the extent of cooperativity of IPTG affecting LacI activity, which followed the *lacI-ZYA* phylogeny (Table S3 and Fig. S3). Consistent with an overall lack of phylogenetic signal in *lac* regulatory parameters, Mantel tests examining the relationship between pairwise strain distance based on genetic relatedness and expression landscapes did not find significant associations (core, *r* = −0.107, *P* = 0.37; accessory, *r* = −0.12, *P* = 0.34; *lacI-ZYA*, *r* = −0.15, *P* = 0.27).

10.1128/mBio.02232-19.3 FIG S3 *P* values of Pagel’s λ tests of phylogenetic signal for regulatory model and regulatory logic parameters. The null hypothesis is the absence of phylogenetic signal, and *P* values below 0.05 are consistent with a parameter evolving neutrally over a particular phylogeny. Tests for phylogenetic signal are shown against phylogenies based on the core genome, the accessory genome, and sequencing encompassing the *lacI-ZYA* region. Download FIG S3, PDF file, 0.1 MB. Copyright © 2019 Phillips et al. 2019 Phillips et al. This content is distributed under the terms of the Creative Commons Attribution 4.0 International license.

10.1128/mBio.02232-19.9 TABLE S3 Pagel’s λ test of phylogenetic signal in parameter variation. Download Table S3, PDF file, 0.1 MB. Copyright © 2019 Phillips et al. 2019 Phillips et al. This content is distributed under the terms of the Creative Commons Attribution 4.0 International license.

### Relationship between regulatory parameters and growth.

**FIG 4:**
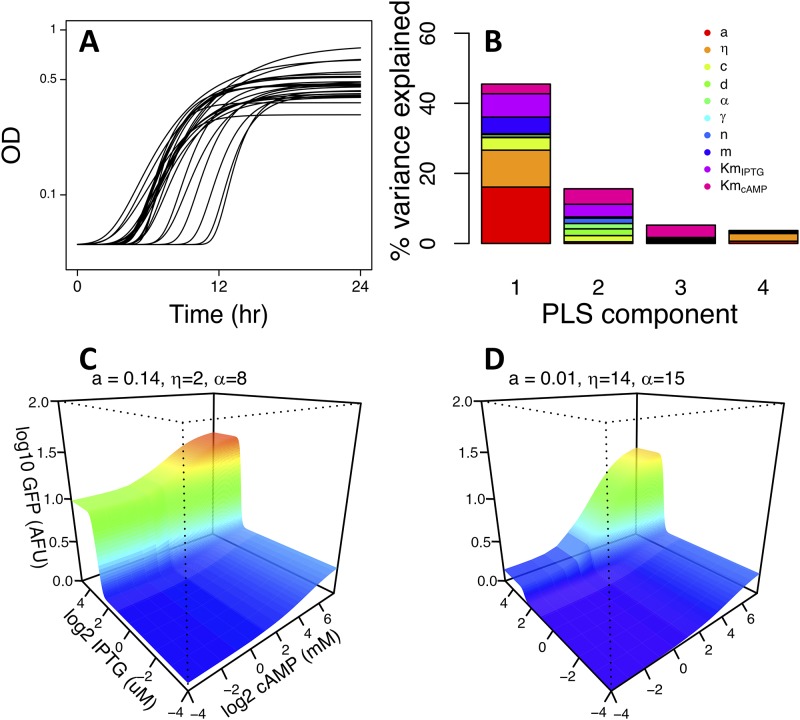
Relationship between regulatory parameters and lag time following transition from a glucose- to lactose-supplemented growth environment. (A) Gompertz fits to growth data of natural isolate strains and hybrid strains containing the *lacI-ZYA* region from a natural isolate strain replacing the same region in the REL606 genetic background. Growth is in lactose following a transition from a day of growth in glucose. (B) Partial least-squares (PLS) regression indicating contribution of regulatory model parameters to the largest four components explaining the variation in lag time. (C and D) Changes in expression landscapes dependent on changing the two parameters*, a* and *η*, that explain the most lag time variation. Parameters are changed between the extremes of their estimated ranges and preserving their negative correlation. Other parameters are as for REL606, except that α is increased in panel D so that maximum expression level is comparable. Panels C and D correspond to landscapes associated with short and long lag times, respectively.

The ideal experiment to test for ecologically meaningful effects of among-strain regulatory differences would be to compare strains that are otherwise identical and determine the fitness consequences of focal regulation phenotypes in a lab, or even natural, environment. The strains we examined are, however, evolutionarily and ecologically divergent and are likely to have growth differences independent of *lac* regulation. Nevertheless, in environments where the effect of *lac* regulation differences are substantial relative to effects of broader background differences, we might see a relationship between these *lac* regulation parameters and a growth phenotype. We chose to focus on the phenotype of lag phase following a transition from growth in glucose to lactose because this transition is likely to depend on the regulatory induction of the *lac* genes, which is a process dependent on the parameters we have measured (Fig. 4A).

To assess the relationship between *lac* regulatory parameters and lag time, we used partial least-squares regression, an approach suited to the analysis of relationships involving a large number of correlated parameters with relatively few data points. This approach was applied to the 23 strains described above, as well as five hybrid strains having *lac* genes moved from natural isolate strains into REL606 (see Materials and Methods). We found that the largest component of the regression explained 45% of the variation in lag time. The largest contributors to this component were the activity of RNA polymerase in the absence of cAMP-CRP (*a*) and its relative stabilization in the presence of cAMP-CRP (*η*), which together accounted for 59% of the component (Fig. 4B). The proportion of lag time variation explained in this analysis was meaningful by comparison to a set of 1,000 permutations in which assignment of estimated lag times to strains was randomized (Fig. S4). Moreover, both the *a* and *η* parameters were individually significantly correlated with lag times (Pearson correlation; *a*, *r* = −0.59, *P* = 0.002; *η*, *r* = 0.54, *P* = 0.005). Expression landscapes illustrating the regulatory influence of observed variation in these parameters (and *α*, which determines the maximum expression) are shown in Fig. 4C and D.

10.1128/mBio.02232-19.4 FIG S4 Permutation tests to assess the significance of variation in lag time explained by partial least-squares regression (Fig. 4). (A and B) Histograms show the percentage of variation in lag time explained by the first PLS component (A) and by the two largest contributors to this component (B) based on 1,000 regressions in which the association between strain names and measured lag times was randomized, while the association between names and regulatory parameters was unchanged. Red arrows indicate corresponding estimates of the actual regression. *P* values are based on the rank ordering of actual estimates against randomized regression outputs. Download FIG S4, PDF file, 0.1 MB. Copyright © 2019 Phillips et al. 2019 Phillips et al. This content is distributed under the terms of the Creative Commons Attribution 4.0 International license.

### Mutual information between regulatory function and genetic polymorphisms.

**FIG 5:**
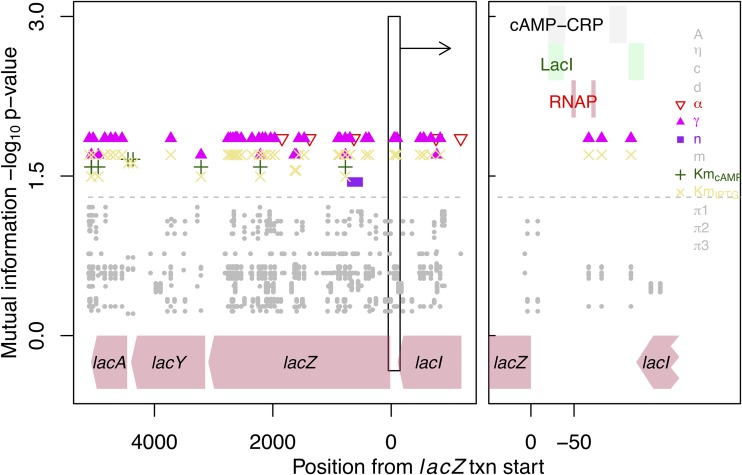
Association between polymorphism in the *lacI-ZYA* region and variation in estimated regulatory parameters. For each parameter, mutual information was estimated between estimates and genetic variation at each site in the genetic region (see Materials and Methods for details). All polymorphic sites are plotted. The dashed line indicates a significance cutoff at a *P* value of 0.05. Gray symbols indicate parameter-polymorphism associations below this cutoff, and colored symbols indicate associations above this cutoff. Only parameters with at least one significant association are colored in the legend. These significant associations primarily affect basal *lac* expression (γ) and the dissociation constant of IPTG from LacI (Km_IPTG_). The left panel presents the entire region considered. The right panel provides higher resolution around the key regulatory area between *lacI-Z* indicated by the box in the left panel. Transcription factor binding sites and the promoter region are indicated in the right panel (binding site information from Regulon DB). RNAP, RNA polymerase; txn, transcription.

We next sought to identify variable sites in known regulatory regions that are correlated with variation in estimated regulatory parameters. We used a measure of mutual information to assess the association between 322 variable sites throughout the *lacI-ZYA* region with variations in estimated regulatory parameters (Fig. 5). This analysis identified a large number of sites associated with regulatory parameters, though significance levels were both generally low and similar across sites for a given parameter, a signature of linkage between genetic variants that are and are not driving regulatory variation. Together, these results suggest that regulatory variation is driven by some combination of a complex genotype-phenotype mapping (e.g., multiple genetic variants may cause similar phenotypic effects, or phenotypes are due to the combined effect of multiple variable sites) and by variable regions outside the one considered here.

### Dependence on genetic background of gene input function.

**FIG 6:**
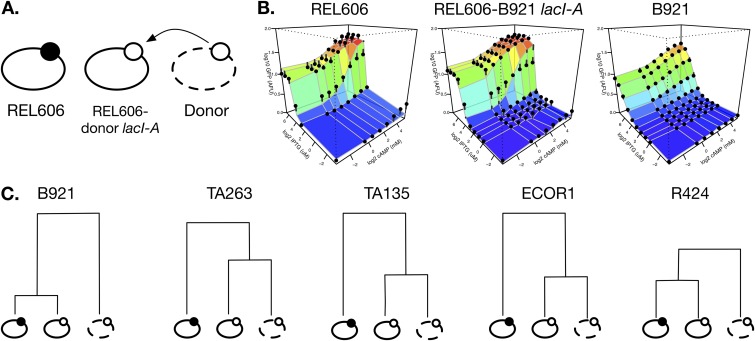
Effect of genetic background on *lac* expression. (A) Schematic of expression comparisons. The *lac* expression profile was obtained from a common reference strain (REL606), different donor natural isolate strains, and a hybrid constructed by swapping the donor strain’s *lacI-ZYA* genes into REL606 (details are in Materials and Methods). (B) Example expression profiles of one comparison set. In this case, the hybrid strain has an expression profile more similar to that of the recipient background strain (REL606) than of the donor (B921). (C) Dendrograms clustering for each of five donors the set of three strains based on Euclidean distances among modeled expression landscapes. The height of dendrograms is scaled to the distance between strains. Expression profiles for each strain are presented in Fig. S5.

To characterize the dependence of *lac* operon regulation on its broader genetic background, we assessed the regulation of different *lac* operons in their native and in a common genetic background. We replaced the *lacI-ZYA* region of REL606 with the corresponding region of five natural isolate strains and determined *lac* expression profiles (Fig. 6 and S5). In general, there was relatively little divergence in profiles, but there were examples of the hybrid strain having *lac* expression more similar to that in the strain comprising the broader genetic background (i.e., REL606), such as in the cross between REL606 and B921, indicating that regulatory elements outside the immediate *lacI-ZYA* region are important in determining its regulation. We also saw examples of the *lac* regulation in the hybrid being more similar to that of the donor strain (e.g., the cross between REL606 and FBGM17), indicating the dominance of local *cis*-regulatory sequences.

10.1128/mBio.02232-19.5 FIG S5 Effect of genetic background on *lac* expression. Expression profile of the lab strain REL606 is presented in the left column (mean of 5 separate profiles). The second column from left presents expression profiles of hybrid strains constructed by adding the *lacI-ZYA* region from a donor natural isolate strain into REL606 (details in Materials and Methods). The second column from right presents the expression profile of the donor natural isolate strain. Dendrograms indicate clustering of each set of three strains (i.e., REL606, REL606 with introduced *lac* region, and natural isolate strain) based on the Euclidean distance of empirically determined expression profiles. Download FIG S5, PDF file, 0.1 MB. Copyright © 2019 Phillips et al. 2019 Phillips et al. This content is distributed under the terms of the Creative Commons Attribution 4.0 International license.

## DISCUSSION

We characterized and compared *lac* regulation of 23 diverse E. coli strains (Fig. 1). We found substantial variation between strains, especially in the degree to which IPTG was individually able to regulate expression to a maximum level (Fig. 3). This variation is consistent with findings of regulatory models that predict that small genetic changes can have large regulatory consequences, revealing that a substantial portion of this potential is realized among natural isolate strains (14). Such regulatory changes can evolve quickly and have ecological consequences (44). Regulatory variation was not well explained by the genetic relatedness of strains, consistent with it being selected rather than evolving neutrally. We also found that a significant part of regulatory variation is likely to depend on factors determined by the genetic background in which the *lac* genes are expressed, as well as on the identity of those genes themselves.

The most variable of the regulatory parameters we examined was the ratio of expression induced by IPTG alone to maximum *lac* expression induced by the presence of both cAMP and IPTG (π2 in Fig. 3). This parameter describes the extent to which *lac* expression depends on the LacI repressor, with less influence by cAMP. One consequence is the possibility that strains where *lac* expression depends less on cAMP, and by extension, the absence of glucose in the environment, might weaken the hierarchy of resource that is determined by the concentrations of preferred resources below which cells switch to catabolism of other alternative resources. The concentration of cAMP has been shown to be critical for determining these concentration crossover points (9). Resources catabolized by genes that were less dependent on cAMP for expression were used preferentially to resources that depended on higher cAMP concentration for their utilization. A previous study of cells evolved in a mix of glucose and lactose sugars found changes in *lac* operon regulation evolved that caused the cells to become more sensitive to the presence of an inducer, consistent with relaxation of the resource use hierarchy (21).

Diversity of *lac* regulation indicates the likelihood of a diversity of regulatory responses to different natural environmental conditions, consistent with previous work finding that different *lac* structural genes can confer different growth responses (4). It is clearly of interest to determine exactly what ecological consequences the different regulatory profiles might have, especially given that profiles were determined under artificial conditions. In practice, however, this is difficult to do because the strains we examine differ in ways other than in their regulation of the *lac* operon, so it is not possible to isolate the influence of *lac* regulatory differences to strain fitness across particular environments. This issue is controlled for among strains we constructed, in which different *lac* genes were transferred to a common background, but here, regulation often differed from that in the donor strains, so that differences in effects cannot be easily interpreted with respect to their donor context. Despite the confounding effect of different backgrounds, we still found a significant relationship between some *lac* regulatory parameters and the transition of our strains from growth in glucose to growth in lactose. This result underlines the potential ecological relevance of the regulatory differences we see.

Several studies have identified natural genetic variation underlying ecologically relevant differences in the regulation of focal genes (4, 36, 45). We found limited indication of an association between genetic polymorphism in the *lacI-ZYA* region and variation in regulatory parameters. Although it is not possible to determine which, if any, of the single-nucleotide polymorphisms (SNPs) we considered might be driving regulatory variation, we note that there were clusters of significant associations between polymorphisms at the end of the *lacZ* and *lacA* genes with the basal level of *lac* reporter expression (determined by γ). There are several possible sources of regulatory variation within these regions. In the end of the *lacZ* gene, there are sites that are responsible for substrate binding, and variation in these sites can affect LacZ catalytic activity and allolactose production (46–48). In *lacA*, associations occur in the stem-loop transcription terminator and in the preceding AT-rich region, suggesting that they might affect transcriptional termination and thereby influence levels of *lacZYA* transcripts. We note as well that the *lac* expression parameters we identify using IPTG and exogenous cAMP may not be realizable in natural environments, for example, because inducer exclusion causes lactose uptake to be more dependent than IPTG uptake on the absence of glucose. To the extent that this is true, some features of the underlying regulatory network are not expected to be directly accessible for selection. In general, however, we interpret the lack of a clear association between polymorphisms and regulatory variation as indicating that most regulatory variation is complex, having a different genetic basis in different strains as well as likely involving the action of several sites, including genes outside the canonical regulatory network.

A substantial portion of the regulatory variation we considered was not explained by patterns of relatedness determined on the basis of core or accessory genomes or of the genes involved in *lac* utilization. Discordant patterns of phenotypic and genetic evolution are consistent either with regulatory parameters varying neutrally at high rates or being selected for in a pattern distinct from that determined by the genetic relatedness of the strains. A previous study found that ecological performance of strains from the same collection used here was not correlated with core or accessory phylogenies, consistent with the possibility that selection might be important (42). This possibility is supported by our finding that regulatory parameters correlated with growth dynamics in at least one environment.

Our finding that the broader genetic background can have substantial influence on the regulation of transferred *lac* operons highlights the importance of noncanonical regulation in determining the expression of *lac* genes. An example of such regulation is the influence of DNA supercoiling on the accessibility of regulatory proteins to the *lac* promoter (26). We note that an influence of the broader background on gene regulation complicates the goals of rational design of regulatory networks, potentially putting a premium on strategies that increase robustness. A strong dependence on genetic background might also lead to greater variation in regulation between strains, increasing the chance that an effective regulatory strategy can be found in changing environments, but also making it less likely that regulation will be successful following horizontal transfer of the *lac* genes to other recipient strains.

In summary, we found that diverse strains of E. coli have different *lac* regulatory profiles, most of which were realized as differences in the form of the regulatory function and of the relative influence of the regulators, cAMP and IPTG, on expression. This variation reveals a wealth of raw material on which selection can act to optimize gene regulation to new environmental challenges. It also poses a challenge to relevant models to be able to explain this diversity of regulation, with some of it coming from outside the canonical regulatory network.

## MATERIALS AND METHODS

### Bacterial strains and strain construction.

Natural isolate strains used as recipients of a *lac* reporter construct were chosen from a collection of 96 strains collected and sequenced as part of a Broad Institute project and obtained from the Michigan State University STEC Center, as well as from strains described in reference 49 (Fig. 1 and Table S1). The genome sequences of the strains were downloaded from the Broad Institute website (https://olive.broadinstitute.org/projects/Escherichia%20coli%20Antibiotic%20Resistance) or obtained by *de novo* Illumina sequencing, as described previously (42). One strain, B156, was included in this work despite being classified as Escherichia albertii. This species lacks a functional LacI repressor and LacY permease and is unable to grow on lactose. Throughout, we include this strain in individual strain descriptions but omit it from the summary data.

The lab strain REL606 was used as the recipient for the transfer of *lacI-ZYA* genes from five natural isolate strains. First, we deleted the corresponding genes in REL606 and replaced them with a chloramphenicol resistance (Cm^r^) gene cassette. To do this, we amplified the chloramphenicol cassette from pKD3 (50) using primers containing 5′ extensions complementary to the REL606 sequence on either side of the *lacI*-*ZYA* genes (forward primer [overlaps the region immediately downstream of *lacA* and pKD3], 5′-GCTGAACTTGTAGGCCTGATAAGCGCAGCGTATCAGGCAATTTTTATAATTGTGTAGGCTGGAGCTGCTTC, and reverse primer [overlaps the region immediately downstream of *lacI* and pKD3], 5′-GCGGTATGGCATGATAGCGCCCGGAAGAGAGTCAATTCAGGGTGGTGAATCATATGAATATCCTCCTTAG). This product was used to transform REL606 containing the red recombineering plasmid pSIM5 (51), and Cm^r^ transformants were selected. These strains had the *lacI-ZYA* gene region replaced by the Cm^r^ gene. In the second step, this replacement strain containing pSIM5 was transformed with the *lacI-ZYA* region amplified from donor natural isolate strains using Phusion Hot Start polymerase (New England BioLabs, MA) (forward primer [overlaps at 39 bases downstream of *lacA*], 5′-AGGCCTGATAAGCGCAGCGT, and reverse primer [overlaps at 44 bases upstream of *lacI*], 5′-TGGCATGATAGCGCCCGGAA). Transformants were selected for incorporation of the incoming DNA by plating on Davis-Mignoli (DM) minimal medium supplemented with thiamine and containing lactose as the sole carbon source. The transformed cells contain *lacI-ZYA* and 44 bases upstream of the *lacI* gene from the donor while maintaining the −35 promoter site of *lacI* from REL606. Sequencing of the junctions between recipient and incoming DNA was performed to confirm the successful incorporation of incoming DNA into the target chromosomal site.

Expression of the *lac* operon was measured using a reporter construct controlled by the P*lac* promoter region, including the O1 and O3 LacI and the primary CRP binding sites (21). This reporter was cloned into a mini-Tn*7* cassette in a suicide vector that was introduced into target strains by conjugation (52). Transposition into the recipient strain *att*Tn*7* site was confirmed by PCR. Although the reporter encodes its own *cis*-regulatory sites and is present at a chromosomal location separate from the native *lac* operon, it does reflect the expression of the native operon because it responds to inducer levels in the cell as a whole, which are determined by expression of the LacY permease encoded by the native operon. Previous work has shown that reporter-driven GFP expression is correlated to native *lac* operon expression, as judged by direct enzymatic assays (13, 21).

### Expression assays.

Regulatory input functions were characterized by measuring the expression of a P*lac*-GFP reporter at different combinations of cAMP and IPTG in DM supplemented with 2,000 μg/ml glucose. This environment was used because glucose inhibits the production of cAMP, allowing measurement of the regulatory input function from as close to the basal level of P*lac*-GFP expression as possible. Strains containing the P*lac*-GFP reporter were preconditioned in DM medium supplemented with 2,000 μg/ml (DM2000) glucose for 24 h and then transferred at a 1:1,000 dilution to the test environments containing combinations of DM2000 supplemented with cAMP and IPTG. cAMP was added at eight concentrations (0, 0.625, 1.25, 2.5, 5, 10, 20, and 40 mM), and IPTG was added at 10 or 6 concentrations (0, 0.78, 1.56, 3.125, 6.25, 12.5, 25, 50, 100, and 200 μM, or 0, 6.25, 12.5, 25, 50, and 100 μM), as noted in the text. Strains were grown in these environments for ∼16 h to an optical density at 450 nm (OD_450_) of ∼0.1 to 0.2, which corresponded to mid-log growth phase, as determined by tracking changes in population OD using a VersaMax spectrophotometer (Molecular Dynamics, CA). An OD of 0.1 reflects approximately six population doublings from the initial inoculum, such that we assume that GFP expression is at steady state and at a level dependent on promoter activity. GFP expression was measured using an Accuri C6 (Becton, Dickinson, NJ) flow cytometer. The analysis pipeline was implemented in R. Expression estimates are presented as arbitrary fluorescence units following subtraction of the fluorescence value of the corresponding strain that did not contain the P*lac*-GFP reporter. In comparisons of gene regulatory functions involving the reference strain (REL606), a natural isolate strain, and a hybrid with the natural isolate *lacI-ZYA* region replacing that of REL606, all compared strains were measured in the same experimental block.

### Phylogeny construction.

Core (shared across all recipient strains) and accessory (shared among a subset of strains) gene regions were identified comparing DNA sequence windows as implemented in Panseq (53). Core regions were defined as regions of 250 bp present in an arbitrary reference strain that were present at a match of >80% identity in all other strains. A phylogeny was built from the core genome by concatenating core regions for each strain and performing a multiple-sequence alignment. Variable sites in this alignment were extracted as an SNP file. We also generated alignments based on the *lacI-ZYA* region alone. The gene region alignment and the core and accessory genomes were used to build phylogenies with which to test for a phylogenetic signal in regulatory parameters estimated from the different test strains. In all cases, PhyML was used to build maximum likelihood trees. For the accessory genome, a binary input file indicating the presence/absence of each accessory gene in each strain was analyzed using default parameters of PARS in PHYLIP (54).

### Growth rate estimation.

Strains were inoculated into LB and grown overnight at 37°C with shaking. A 2-μl aliquot of each culture was transferred to each of three wells in a microtiter plate containing 200 μl DM200 glucose medium. Following 24 h of incubation at 37°C with shaking, a 1:100 dilution was made into another microtiter plate containing the same medium. After a second 24-h incubation, another 1:100 dilution was made into a microtiter plate containing DM1500 lactose, and the new plate was incubated in a VersaMax plate spectrophotometer. OD_450_ readings and 3-s shaking periods were carried out every 3 min for 24 h. A custom script was used to fit a modified Gompertz growth function to the resulting growth data (55). Growth parameters for each strain were estimated as the average of estimates for individual replicates weighted by the quality of each fit. In the modified Gompertz function, the parameter best interpreted as lag time, λ, corresponds to the time taken for a population to reach its maximum growth rate.

### Model and statistical analyses.

All analyses were carried out using R (version 3.4.3) (56). Regulatory input functions were analyzed in two stages. First, the optim function was used to estimate parameters of a simple model incorporating key features of *lac* regulation that best fit observed GFP expression at each combination of cAMP-IPTG concentrations (equation 1 in reference 14). A detailed outline of this model is presented in the supplemental material. Briefly, it includes terms that describe CRP activity (fraction bound to cAMP) (*A*), cAMP-CRP binding cooperativity (*n*), LacI activity (fraction not bound to IPTG) (*R*), LacI-IPTG binding cooperativity (*m*), affinity to binding sites of RNA polymerase in the absence of cAMP-CRP (*a*), cAMP-CRP (*d*), and LacI (*d*), the effect of cAMP-CRP binding on RNA polymerase binding stability (η), and maximum (α) and basal (γ) expression rates. The model omits some molecular details, such as DNA looping stabilized by bound LacI tetramers, that are known to influence *lac* expression (15). Nevertheless, for all strains, the fitted models captured a substantial portion of the overall expression variation (root mean square error [RMSE] of the fitted models was low relative to overall variation in expression [mean, 0.106; standard deviation, 0.055]).

Estimates of each model parameter were used to predict an idealized regulatory function that characterized the individual and combined effect of IPTG and cAMP on *lac* expression (14). Following previous work, we used an artificial inducer, IPTG, to manipulate LacI activity. IPTG is not metabolized, allowing concentrations to be maintained through cell growth and reducing potential feedback between inducer concentration and cell growth rates (20). The resulting expression profiles will probably differ from those that would be seen if the natural lactose inducer was used. One reason for this is that IPTG can passively diffuse into cells, allowing a baseline intracellular concentration independent of the LacY permease and reducing the influence of inducer exclusion, a posttranslational regulation mechanism through which glucose indirectly reduces the activity of the LacY permease (15, 18, 57). Reduced inducer exclusion has the effect of allowing LacI-mediated negative regulation and cAMP-CRP-mediated positive regulation to be controlled independently so that all combinations of their activity can be measured even when some combinations may not be accessible during growth in environments containing only natural inducers. We note that many of the analyses we present focus on *lac* expression occurring at saturating inducer concentrations, where LacY-independent uptake of IPTG is not expected to have any additional regulatory effect. Supporting this, we observed good correspondence between *lac* expression estimates using high levels of IPTG and methyl-β-d-thiogalactoside (TMG), an inducer that depends on LacY for import (Fig. S6). We also find a significant correlation between expression levels during growth of strains in (i) glycerol, an environment supporting the production of high levels of endogenous cAMP, and in glucose supplemented with exogenous cAMP, and (ii) lactose and glucose supplemented with cAMP and IPTG (Fig. S6).

10.1128/mBio.02232-19.6 FIG S6 Comparison of assay and inducer types on inferred *lac* expression. Expression values indicated on the horizontal axes are as reported in the text and are inferred from fluorescence derived from a P*lac*-*gfp* reporter construct. Strains were grown in glucose supplemented with the artificial inducer IPTG (200 μM) and/or exogenously supplied cAMP (40 mM), as noted on the axis titles. Expression profiles reported on the vertical axes are inferred from Miller assays (58) in environments using the natural inducers glycerol (which supports high intracellular cAMP) and lactose, and the artificial inducer TMG (200 μM), which depends on LacY for uptake. Combinations of these inducers are chosen to manipulate LacI and CRP regulation in a way that recapitulates as closely as possible effects using IPTG and exogenous cAMP (glucose + cAMP and glycerol environments, high cAMP-low IPTG; glucose + IPTG and glucose + TMG, low cAMP-high IPTG; glucose + cAMP + IPTG and lactose, high cAMP-high IPTG). Pearson correlation analyses are reported on each plot. Miller assays measure LacZ enzyme activity directly, providing a control for expression inferred from the fluorescent reporter construct used in our main set of experiments. Download FIG S6, PDF file, 0.1 MB. Copyright © 2019 Phillips et al. 2019 Phillips et al. This content is distributed under the terms of the Creative Commons Attribution 4.0 International license.

Regulatory parameter estimates were tested for an association with genetic variation in the *lacI-ZYA* region of 18 of the strains for which expression and regulatory parameter information was available (the sequence of the entire *lacI-ZYA* region was not available for strains B156, B1167, TA135, TA263, and H413). Alignment of this region included 6,298 bases, of which 322 sites were polymorphic. The function BUS in the BUS package was used to determine the association between estimated *lac* expression parameters and polymorphism. The mutual information between these variables was determined, and significance was estimated using a permutation approach to correct for testing over multiple sites (using option method = 2).

Tests for phylogenetic signal were performed using the function phylosig in the Phytools package. The functions pd.calc and pd.bootstrap in the package Caper were used to test whether the strains we used were representative of the diversity present in our larger collection of 96 strains (Fig. S1). To do this, we compared the distance separating the strains used here to a distribution of distances between 1,000 randomly chosen sets of the same number of strains from the 96 sequenced strains contained in our overall phylogeny. Comparisons between strain expression descriptions (model parameterization, logic phenotypes, and the complete expression landscape) were performed using nonparametric Mantel tests, as implemented in the Ecodist package.

### Data availability.

All relevant data and method scripts have been archived at Dryad (https://doi.org/10.5061/dryad.8cz8w9gk9).
