# Supplementary figures and images for: Diversity in lac Operon Regulation among Diverse Escherichia coli Isolates Depends on the Broader Genetic Background but Is Not Explained by Genetic Relatedness
Source: mBio. 2019 Nov 12;10(6):e02232-19. doi: 10.1128/mBio.02232-19 (PMC6851279; doi:10.1128/mBio.02232-19)

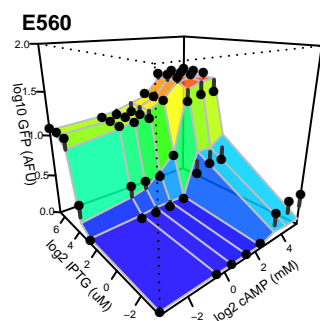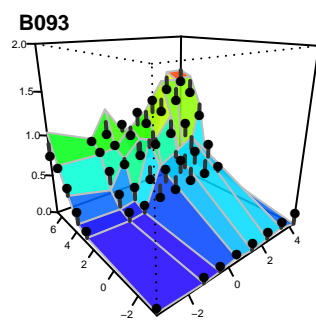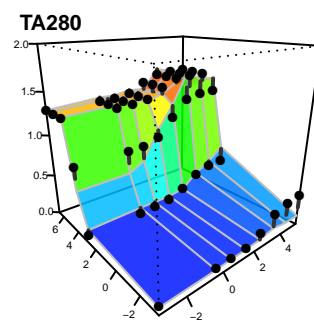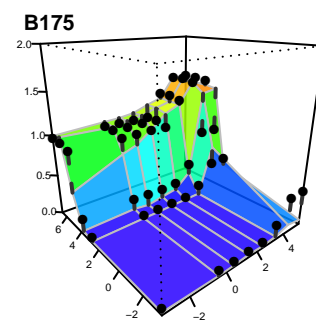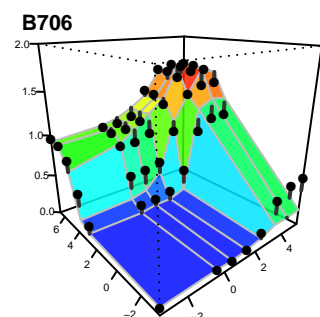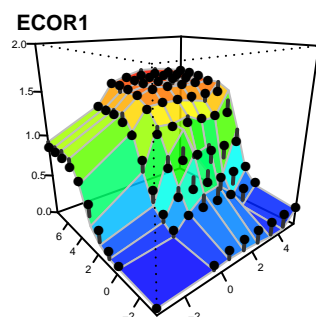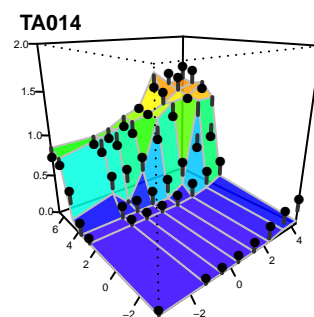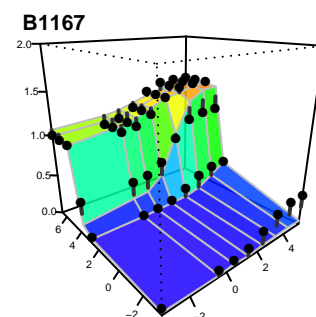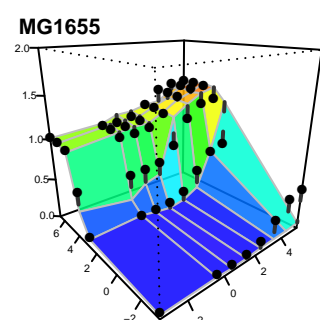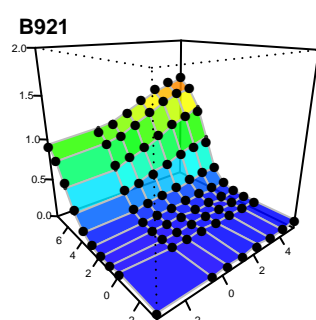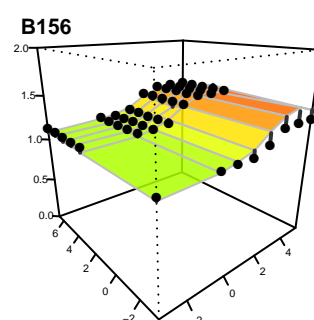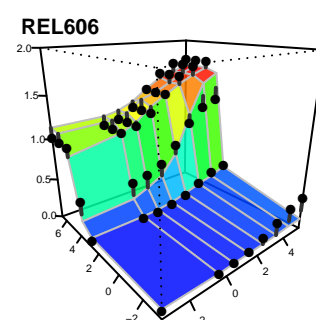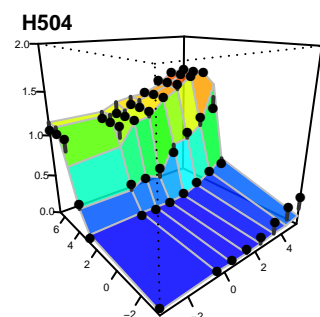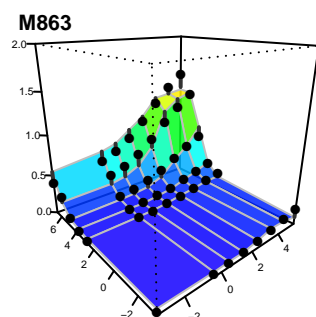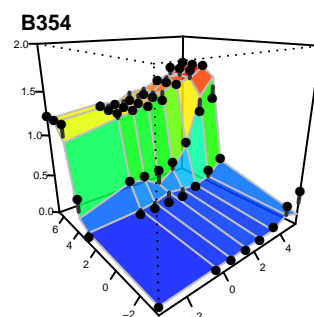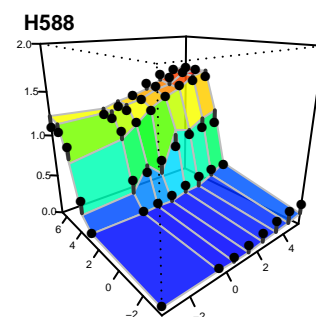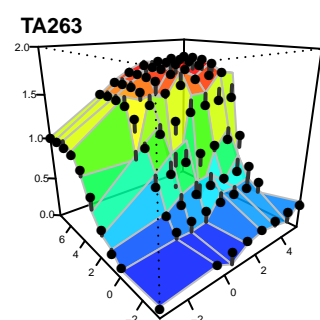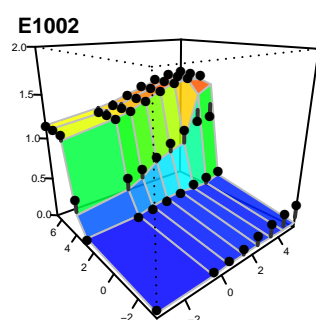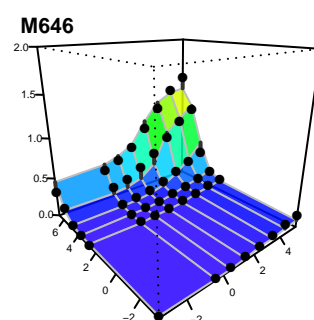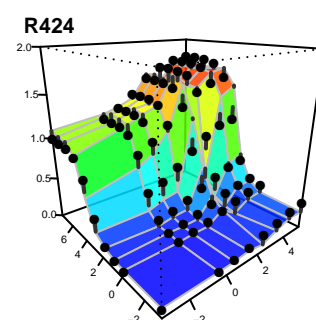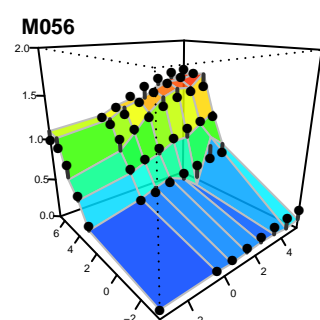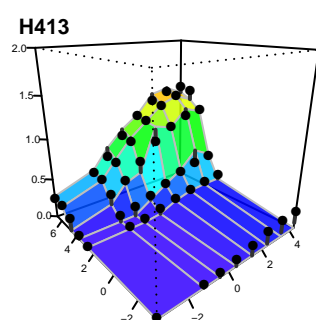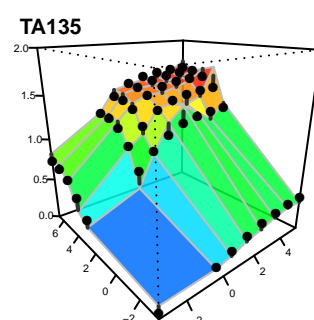

Supplement: FIG S2 [file mBio.02232-19-sf002.pdf]

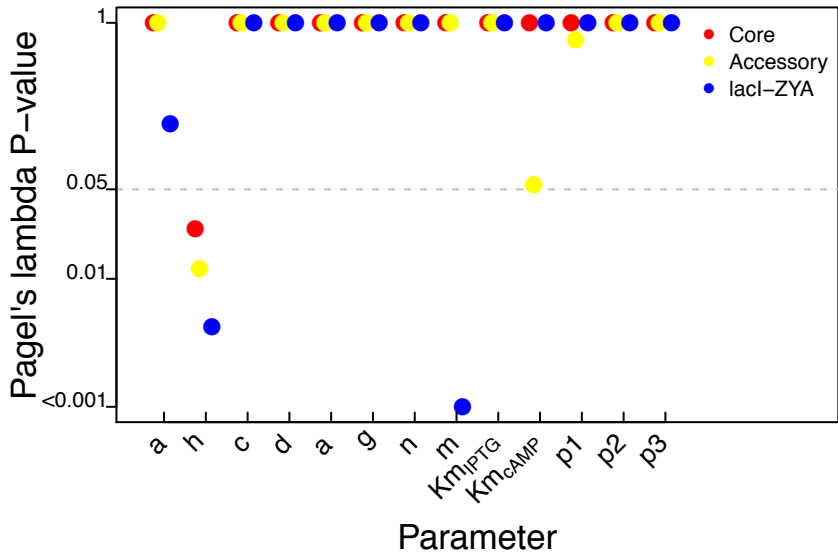

Supplement: FIG S3 [file mBio.02232-19-sf003.pdf]

**A**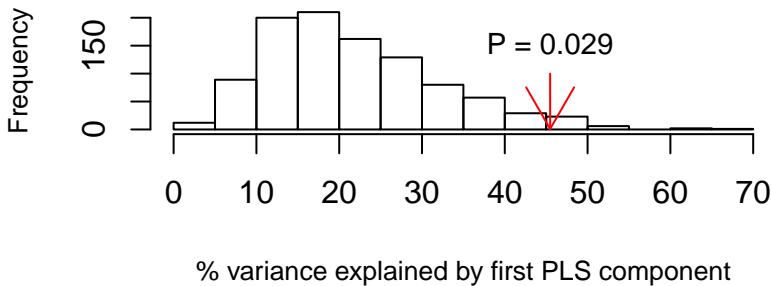**B**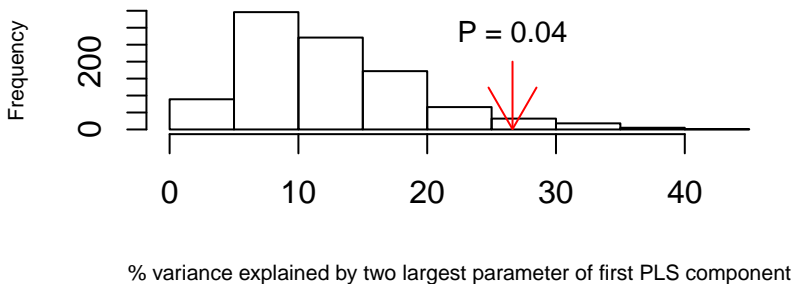

Supplement: FIG S4 [file mBio.02232-19-sf004.pdf]

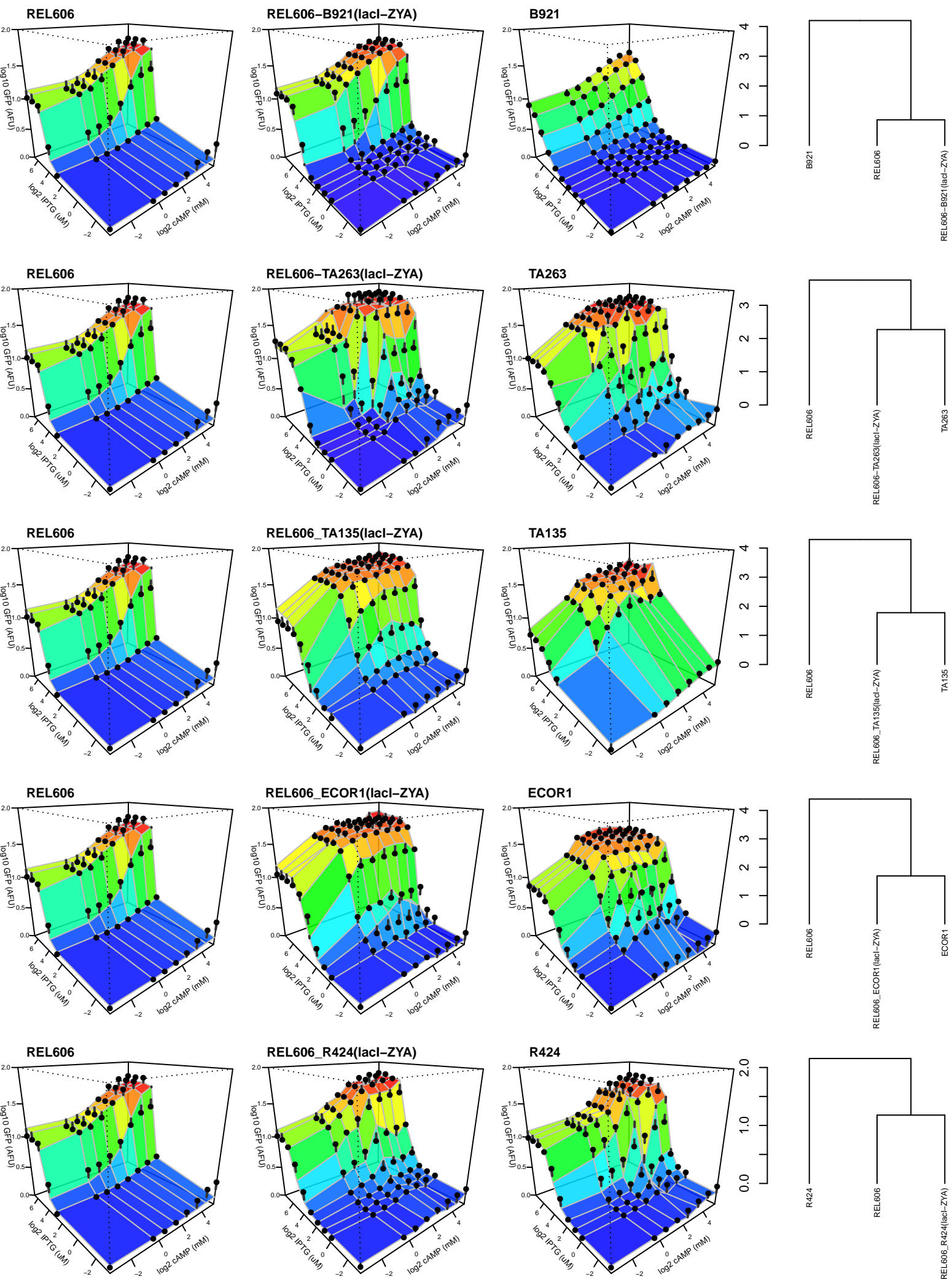

Supplement: FIG S5 [file mBio.02232-19-sf005.pdf]

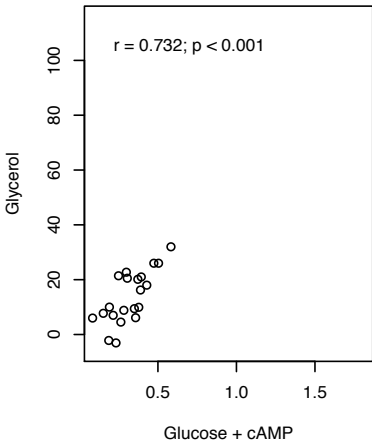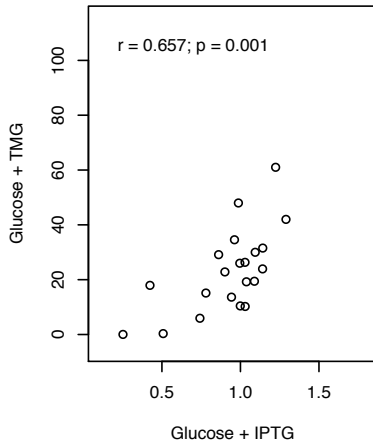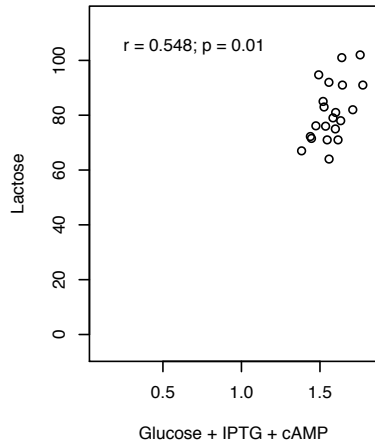

Supplement: FIG S6 [file mBio.02232-19-sf006.pdf]
