# Supplementary material for: Diversity in lac Operon Regulation among Diverse Escherichia coli Isolates Depends on the Broader Genetic Background but Is Not Explained by Genetic Relatedness
Source: mBio. 2019 Nov 12;10(6):e02232-19. doi: 10.1128/mBio.02232-19 (PMC6851279; doi:10.1128/mBio.02232-19)
Supplement: TABLE S1 [file mBio.02232-19-st001.pdf]

Table S1. Strains used in this study.

Strain ID[a]	Alternative ID	Obtained from	Genome sequence
B093	TW15931	Broad Institute via MSU STEC center	Broad[b]
B156	TW15934	Broad Institute via MSU STEC center	
B175	TW15935	Broad Institute via MSU STEC center	Broad
B354	TW15938	Broad Institute via MSU STEC center	Broad
B706	TW15943	Broad Institute via MSU STEC center	Broad
B921	TW15945	Broad Institute via MSU STEC center	Broad
B1167	TW15933	Broad Institute via MSU STEC center	
E560	TW15955	Broad Institute via MSU STEC center	Broad
E1002	TW15946	Broad Institute via MSU STEC center	Broad
TA135	FBGM4	F.B.-G. Moore	Broad
TA263	FBGM10	F.B.-G. Moore	
ECOR1	FBGM17	F.B.-G. Moore	Wang et al. 2016
H413	TW15974		Broad
H504	TW15981	Broad Institute via MSU STEC center	Broad
H588	TW15982	Broad Institute via MSU STEC center	Broad
M056	TW15990	Broad Institute via MSU STEC center	Broad
M646	TW15993	Broad Institute via MSU STEC center	Broad
M863	TW15995	Broad Institute via MSU STEC center	Broad
MG1655		CGSC	NCBI: NZ_CP027060
R424	TW15997	Broad Institute via MSU STEC center	Broad
REL606		R. E. Lenski	Jeong et al. 2009
TA014	TW16005	Broad Institute via MSU STEC center	Broad
TA280	TW16018	Broad Institute via MSU STEC center	Broad

[a] ID from Moore and Woods (2006) or MSU STEC center documentation.

[b] Broad genome sequences downloaded from:

http://www.broadinstitute.org/annotation/genome/escherichia_antibiotic_resistance/MultiDownloads.html

1. Wang Y, Díaz Arenas C, Stoebel DM, Flynn K, Knapp E, Dillon MM, Wünsche A, Hatcher P, Moore FB-G, Cooper VS, Cooper TF. 2016. Benefit of transferred mutations is better predicted by the fitness of recipients than by their ecological or genetic relatedness. Proc Natl Acad Sci U S A 113(18):5047–52.

2. Jeong H, Barbe V, Lee CH, Vallenet D, Yu DS, Choi S-H, Couloux A, Lee S-W, Yoon SH, Cattolico L, Hur C-G, Park H-S, Ségurens B, Kim SC, Oh TK, Lenski RE, Studier FW, Daegelen P, Kim JF. 2009. Genome Sequences of *Escherichia coli* B strains REL606 and BL21 (DE3). J Mol Biol 394(4):644–52.

3. Moore FB-G, Woods R. 2006. Tempo and constraint of adaptive evolution in *Escherichia coli* (Enterobacteriaceae, Enterobacteriales). Biological Journal of the Linnean Society. Oxford University Press 88(3):403–11.
