# Supplementary material for: Diversity in lac Operon Regulation among Diverse Escherichia coli Isolates Depends on the Broader Genetic Background but Is Not Explained by Genetic Relatedness
Source: mBio. 2019 Nov 12;10(6):e02232-19. doi: 10.1128/mBio.02232-19 (PMC6851279; doi:10.1128/mBio.02232-19)
Supplement: FIG S1 [file mBio.02232-19-sf001.pdf]

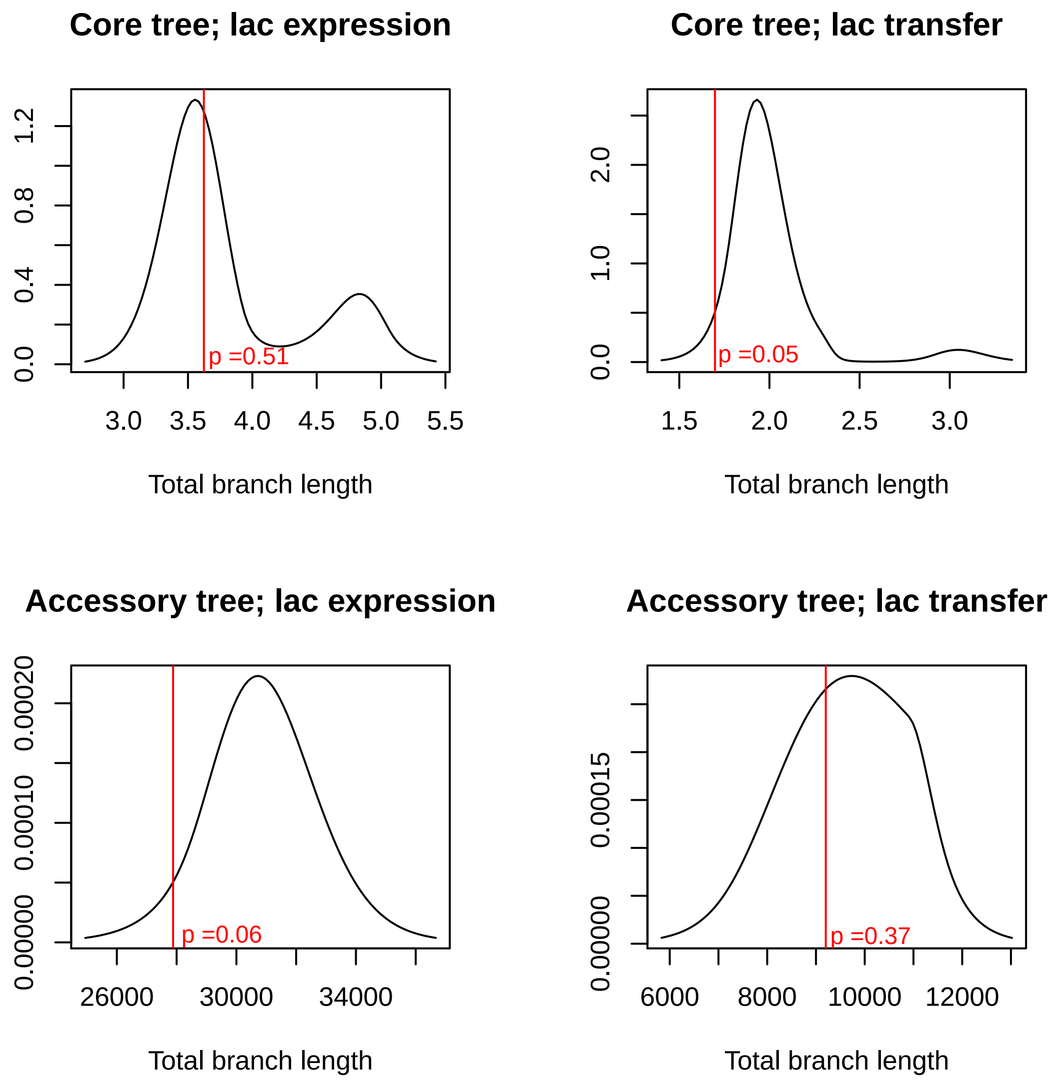
Core tree; lac expression
1.2
0.8
0.4
0.0
p =0.51
3.0
3.5
4.0
4.5
5.0
5.5
Total branch length
Core tree; lac transfer
2.0
1.0
0.0
p =0.05
1.5
2.0
2.5
3.0
Total branch length
Accessory tree; lac expression
0.00020
0.00010
0.00000
p =0.06
26000
30000
34000
Total branch length
Accessory tree; lac transfer
0.00015
0.00000
p =0.37
6000
8000
10000
12000
Total branch length
