# Supplementary material for: Diversity in lac Operon Regulation among Diverse Escherichia coli Isolates Depends on the Broader Genetic Background but Is Not Explained by Genetic Relatedness
Source: mBio. 2019 Nov 12;10(6):e02232-19. doi: 10.1128/mBio.02232-19 (PMC6851279; doi:10.1128/mBio.02232-19)
Supplement: TEXT S1 [file mBio.02232-19-s0001.docx]

**Supplementary Information**

**Model.** Promoter activity at the *lac* operon was characterized based on the steady-state binding of the LacI and CRP regulators using the model and approach presented by (1,2). Promoter activity was calculated as:

$$P=\left( \alpha-\gamma\right)\frac{a\left( 1+\eta.d.A \right)}{1+a+\left( a.\eta+1 \right)d.A+c.R}+\gamma$$

where activity of the CRP activator, assessed as the fraction of CRP bound to cAMP, is A = *X^n^*/(1+*X^n^*), in which *X* = [cAMP]/K_cAMP_ (i.e., cAMP concentration in units of its dissociation constant for CRP). Cooperativity of cAMP binding to CRP is given by the Hill coefficient, *n*. Similarly, the fraction of active LacI repressor not bound to the IPTG inducer is R = 1/(1+*Y^m^*), in which *Y* = [IPTG]/K_IPTG_ (i.e., IPTG concentration in units of its dissociation constant for LacI). Cooperativity of IPTG binding with LacI is given by the Hill coefficient, *m*. The binding affinity of regulators to DNA binding sites is given by parameters *a*, *c*, and *d*. *a* = [RNAp]/K_p_ is the concentration of RNAp (RNA polymerase) in units of its dissociation constant to the free promoter (i.e., CRP is not bound). *c* = [LacI]/K_R_ is the concentration of LacI repressor in units of its dissociation constant with its binding sites. *d* = [CRP]/K_A_ is the concentration of CRP is units of its dissociation constant with its binding site in the *lac* operon *cis*-regulatory region. Stabilization of RNAp by the presence of CRP is given by the ratio of its dissociation constants in the presence and absence of CRP: η = Kp/Kcp. The terms α and γ give maximum and basal transcript rates, respectively.

1. Setty Y, Mayo AE, Surette MG, Alon U. 2003. Detailed map of a cis-regulatory input function. Proc Natl Acad Sci U S A 100(13):7702–7.
2. Mayo AE, Setty Y, Shavit S, Zaslaver A, Alon U. 2006. Plasticity of the *cis*-regulatory input function of a gene. PLoS Biol 4(4):e45–7.
