# Supplementary material for: Diversity in lac Operon Regulation among Diverse Escherichia coli Isolates Depends on the Broader Genetic Background but Is Not Explained by Genetic Relatedness
Source: mBio. 2019 Nov 12;10(6):e02232-19. doi: 10.1128/mBio.02232-19 (PMC6851279; doi:10.1128/mBio.02232-19)
Supplement: TABLE S2 [file mBio.02232-19-st002.pdf]

Table S2. Model parameterization for natural isolate and lab strains used in this study.

Strain	*a*	η	*c*	*d*	α	γ	*n*	*m*	K_{IPTG}	K_{cAMP}	$\pi 1$	$\pi 2$	$\pi 3$
B1167	0.12	1.69	14.24	3.29	9.02	0.00	2.45	9.28	6.17	9.98	0.05	0.69	0.26
B156	0.00	16.79	7.26	11.36	12.82	1.11	4.58	3.98	6.77	1.92	0.78	0.79	0.92
B175	0.14	1.81	13.53	3.46	8.10	0.00	11.21	7.06	11.09	11.35	0.05	0.66	0.29
B354	0.27	1.61	20.17	4.32	5.65	0.01	2.89	59.23	17.80	12.08	0.05	0.74	0.28
B706	0.12	2.03	13.28	5.03	9.16	0.00	3.57	2.44	7.91	4.45	0.04	0.59	0.36
B921	0.07	3.88	40.74	0.35	14.12	0.03	1.37	1.65	4.50	4.38	0.03	0.51	0.05
E1002	0.15	1.72	14.44	1.78	8.61	0.00	1.31	14.21	6.55	10.81	0.05	0.73	0.19
E560	0.11	1.55	36.34	9.53	12.12	0.00	5.71	7.31	7.97	8.55	0.02	0.70	0.25
TA263	0.11	3.33	10.66	0.70	9.81	0.00	0.94	1.86	1.08	1.44	0.05	0.56	0.16
ECOR1	0.10	3.70	11.18	0.53	9.61	0.00	3.58	1.57	0.69	1.12	0.05	0.56	0.14
TA135	0.08	3.17	10.94	1.96	9.79	0.00	1.26	1.46	1.51	1.60	0.04	0.46	0.26
M863	0.04	8.26	13.89	1.02	10.73	0.00	0.97	2.71	17.93	14.82	0.02	0.25	0.15
MG1655	0.12	1.57	13.51	5.29	9.34	0.00	2.69	4.16	11.60	6.62	0.05	0.71	0.36
R424	0.11	2.85	12.47	1.21	9.67	0.00	1.24	2.50	5.86	3.02	0.04	0.55	0.18
REL606	0.14	2.41	13.33	2.10	8.13	0.00	1.70	9.16	10.09	9.89	0.05	0.57	0.23
TA280	0.14	1.32	13.68	2.69	10.24	0.00	2.97	7.95	6.86	8.57	0.06	0.83	0.24
B093	0.09	15.52	11.21	0.08	9.91	0.00	1.05	1.73	4.15	5.00	0.05	0.70	0.25
H413	0.02	10.07	13.12	1.36	10.64	0.00	1.23	3.64	2.67	10.88	0.01	0.18	0.17
H504	0.13	1.74	15.92	3.25	9.28	0.00	1.75	15.07	11.73	10.98	0.05	0.68	0.24
H588	0.14	2.09	12.09	1.03	8.85	0.00	1.56	3.94	5.10	8.04	0.06	0.69	0.17
M056	0.12	3.46	10.81	0.49	9.33	0.00	0.96	2.05	3.65	4.06	0.06	0.60	0.14
M646	0.03	10.72	17.67	0.86	12.79	0.00	1.07	2.72	14.09	17.61	0.01	0.20	0.10
TA014	0.08	3.10	13.44	2.17	9.49	0.00	1.69	3.25	6.35	10.13	0.03	0.46	0.2

Parameters determining activity of the *lac* promoter were estimated by fitting the regulatory model described in SI (from Mayo et al. 2006 (1)). The phenotypic parameters $\pi 1$, $\pi 2$ and $\pi 3$ give the predicted ratio of expression at no induction, high-IPTG_low-cAMP, and low-IPTG_high-cAMP to expression at maximum cAMP and IPTG levels, respectively.

1. Mayo AE, Setty Y, Shavit S, Zaslaver A, Alon U. 2006. Plasticity of the cis-regulatory input function of a gene. PLoS Biol 4(4):e45–7.
