# Supplementary material for: Diversity in lac Operon Regulation among Diverse Escherichia coli Isolates Depends on the Broader Genetic Background but Is Not Explained by Genetic Relatedness
Source: mBio. 2019 Nov 12;10(6):e02232-19. doi: 10.1128/mBio.02232-19 (PMC6851279; doi:10.1128/mBio.02232-19)
Supplement: TABLE S3 [file mBio.02232-19-st003.pdf]

Table S3. Pagel's λ test of phylogenetic signal in parameter variation

	Phylogeny		
Parameter	Core	Accessory	*lacI-ZYA*
a			0.162
η	0.024[a]	0.012	0.004
c			
d			
α			
γ			
n			
m			<0.001
Km_{cAMP}			
Km_{IPTG}		0.055	
$\pi1$		0.733	
$\pi2$			
$\pi3$			

[a]P-values <0.05 are interpreted as a significant deviation from the null model that parameter values are distributed randomly over the phylogeny

Note: For clarity, P-values estimated as 1.0 are omitted
